# Supplementary material for: Metal Complex Formation and Anticancer Activity of Cu(I) and Cu(II) Complexes with Metformin
Source: Molecules. 2021 Aug 5;26(16):4730. doi: 10.3390/molecules26164730 (PMC8401132; doi:10.3390/molecules26164730)
Supplement: Supplementary file 1 [file molecules-26-04730-s001.zip › molecules-1313474-supplementary.pdf]

SUPPLEMENTARY MATERIAL

**Metformin, Cu(II)/Metformin and Cu(I)/Metformin complexes antitµMoral activity.**

*Sherin A. Abdelrahman[1], Mawadda Alghrably[2], Marcello Campagna[3], Charlotte A. E. Hauser*,[1], Mariusz Jaremko*,[2] and Joanna I. Lachowicz*,[3]*

[1] *Laboratory for Nanomedicine, Division of Biological and Environmental Science and Engineering, King Abdullah University of Science and Technology, Thuwal 23955-6900, Saudi Arabia; sherin.abdelrahman@kaust.edu.sa*

[2] *Division of Biological and Environmental Sciences and Engineering (BESE), King Abdullah University of Science and Technology (KAUST), Thuwal 23955-6900, Saudi Arabia; mawadda.alghrably@kaust.edu.sa*

[3] *Department of Medical Sciences and Public Health, University of Cagliari, Policlinico Universitario, I-09042 Monserrato, Italy*

## Table of contents

**Figure S1.** ESI-MS spectrµM of metformin and Cu(II)/Metformin complexes. Enlargement boxes: Comparison of signals of Copper/(metformin (=L) complexes with simulated spectra (Down).

**Figure S2.** X-ray structures AJUHUJ (A), HIBPOX (B), HIHDUX (C), EFIXµM (D) and ETOFOI (E) of Cu(II)/Metformin complexes in different experimental conditions. Data available in CCDS database.

**Figure S3.** 1HNMR spectrµM and proton assigned of GSH according to SDBS database (https://sdbs.db.aist.go.jp/sdbs/cgi-bin/cre_index.cgi).

**Figure S4.** $^{1}$H NMR prediction of metformin by Chemdraw.

**Figure S5.** Anticancer effect of metformin on colorectal cancer (SW1222) cells. (A) Live/dead staining of SW1222 cells treated with different concentrations of metformin. The fluorescent images show live cells (green) and dead cells (red). (B) High resolution confocal images of SW1222 showing actin fibers organization (red) and nucleus (blue). Scale bar (100 µm).

**Figure S6.** Anticancer effect of metformin on leukemic cell line (K562). (A) Live/dead staining of K562 cells treated with different concentrations of metformin. The fluorescent images show live cells (green) and dead cells (red). (B) High resolution confocal images of K652 showing actin fibers organization (red) and nucleus (blue). Scale bar (100 µm).

**Figure S7.** Anticancer effect of metformin on MDR leukemic cell line (K562-200). (A) Live/dead staining of K562-200 cells treated with different concentrations of metformin. The fluorescent images show live cells (green) and dead cells (red). (B) High resolution confocal images of K652-200 showing actin fibers organization (red) and nucleus (blue). Scale bar (100 µm).

**Figure S8.** Anticancer effect of metformin on SW1222 cells in the presence and absence of copper sulfate. Scale bar (100 µM). The fluorescent images show cells shrinking and rounding with increasing concentrations of metformin with complete disappearance of actin fibers at the highest concentration used.

**Figure S9.** Anticancer effect of metformin on K562 cells in the presence and absence of copper sulfate. Scale bar (100 µM). The fluorescent images show cells shrinking and rounding with increasing concentrations of metformin with complete disappearance of actin fibers at the highest concentration used.

**Figure S10.** Anticancer effect of metformin on K562-200 cells in the presence and absence of copper sulfate. Scale bar (100 µM). The fluorescent images show cells shrinking and rounding with increasing concentrations of metformin with complete disappearance of actin fibers at the highest concentration used.

**Figure S11.** Effect of copper on the anticancer properties of metformin. Comparison between the effect on cancer cells of 10 mM (left) and 50 mM (right) metformin in the absence and presence of different concentrations of copper sulfate and the effect of copper sulfate on the same cells in the absence of metformin. (error bars different color)

**Table S1:** Effect of Cu(II) on the anticancer properties of metformin.

**Figure S12.** X-ray structures of Cu(II)/GSSG complex [1]. Data available in CCDS database (https://www.ccdc.cam.ac.uk/structures/).

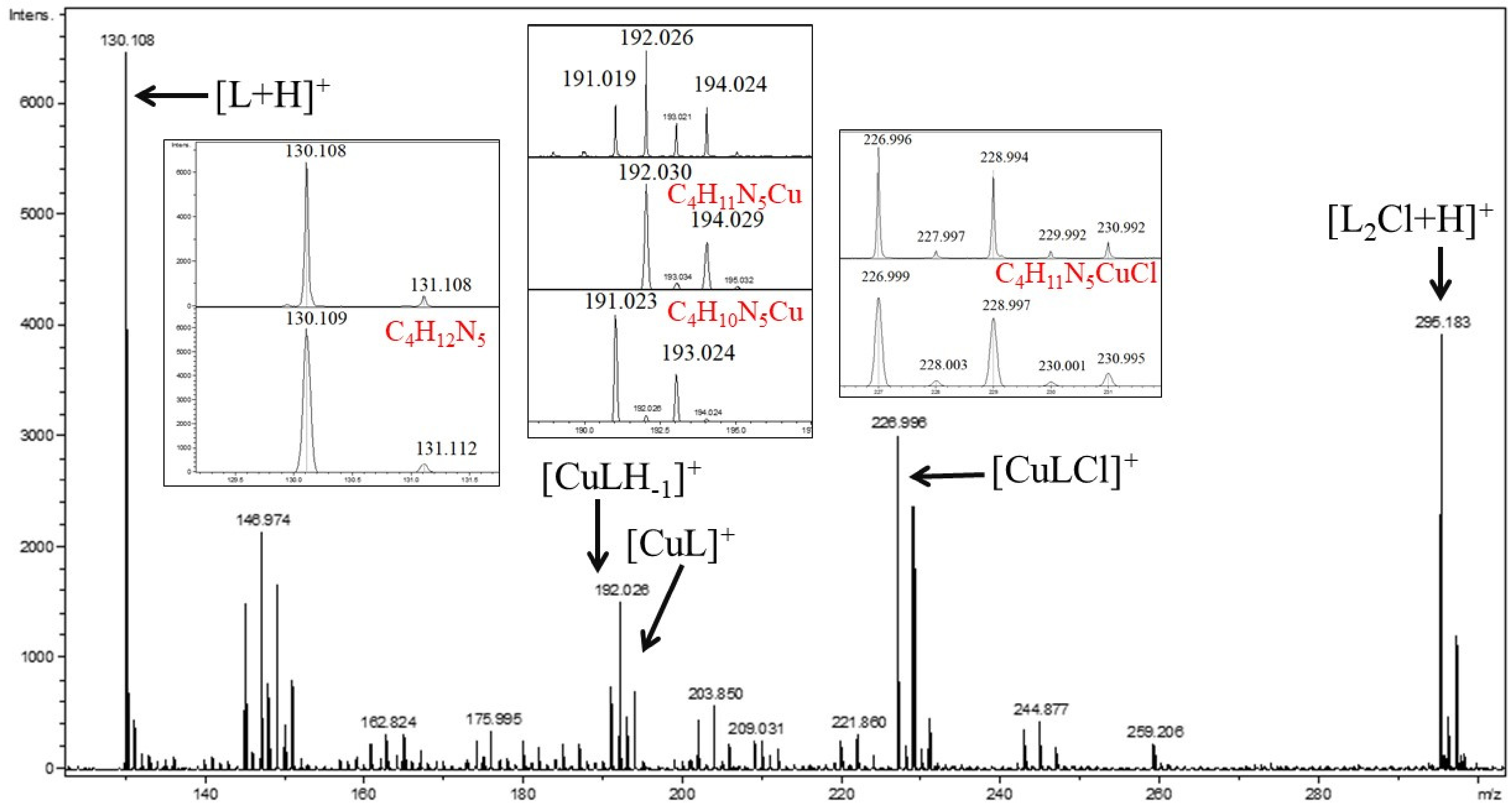


**Figure S1.** ESI-MS spectrµM of metformin and Cu(II)/Metformin complexes. Enlargement boxes: Comparison of signals of Copper/(metformin (=L) complexes with simulated spectra (Down).

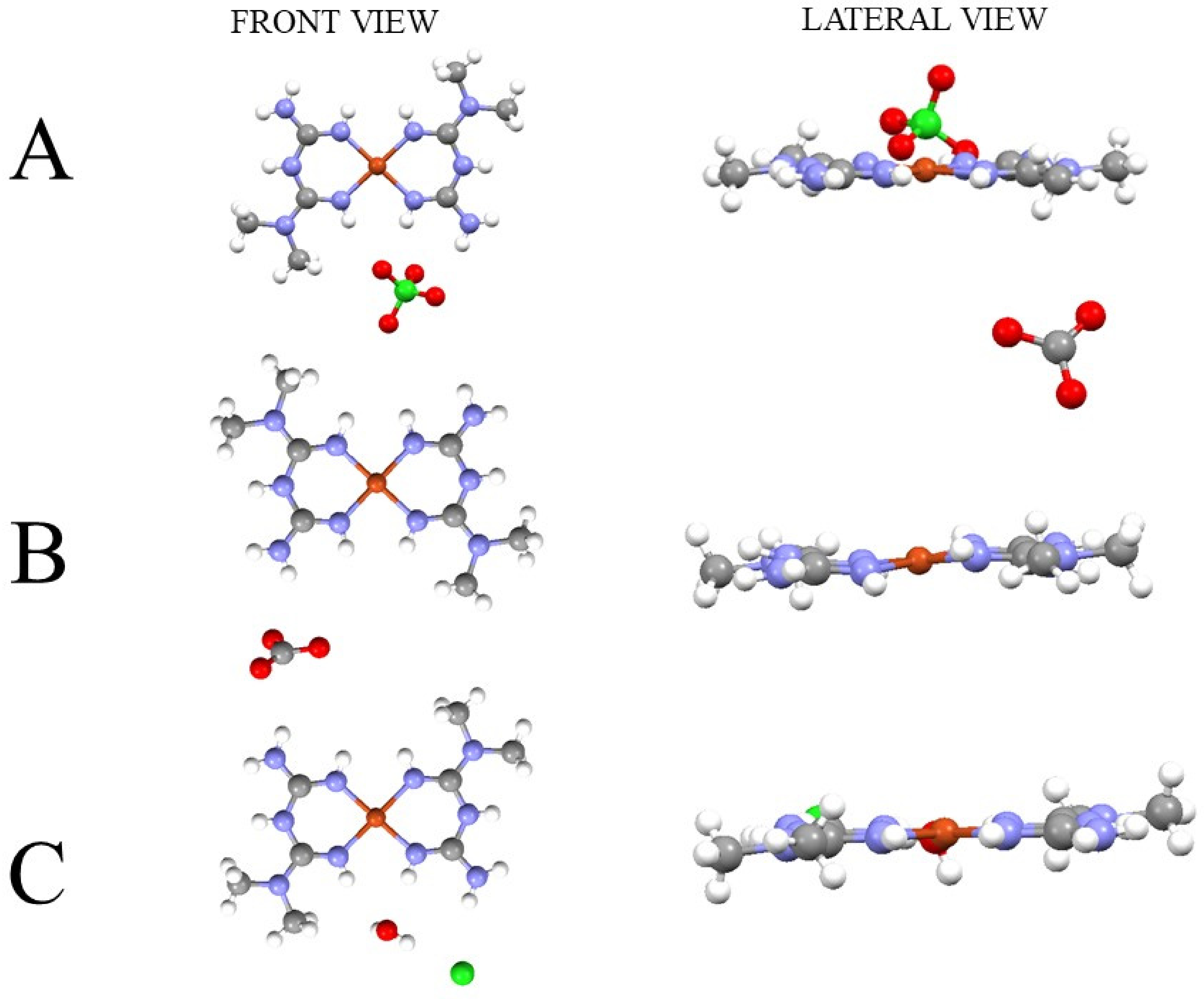
FRONT VIEW
LATERAL VIEW
A
B
C

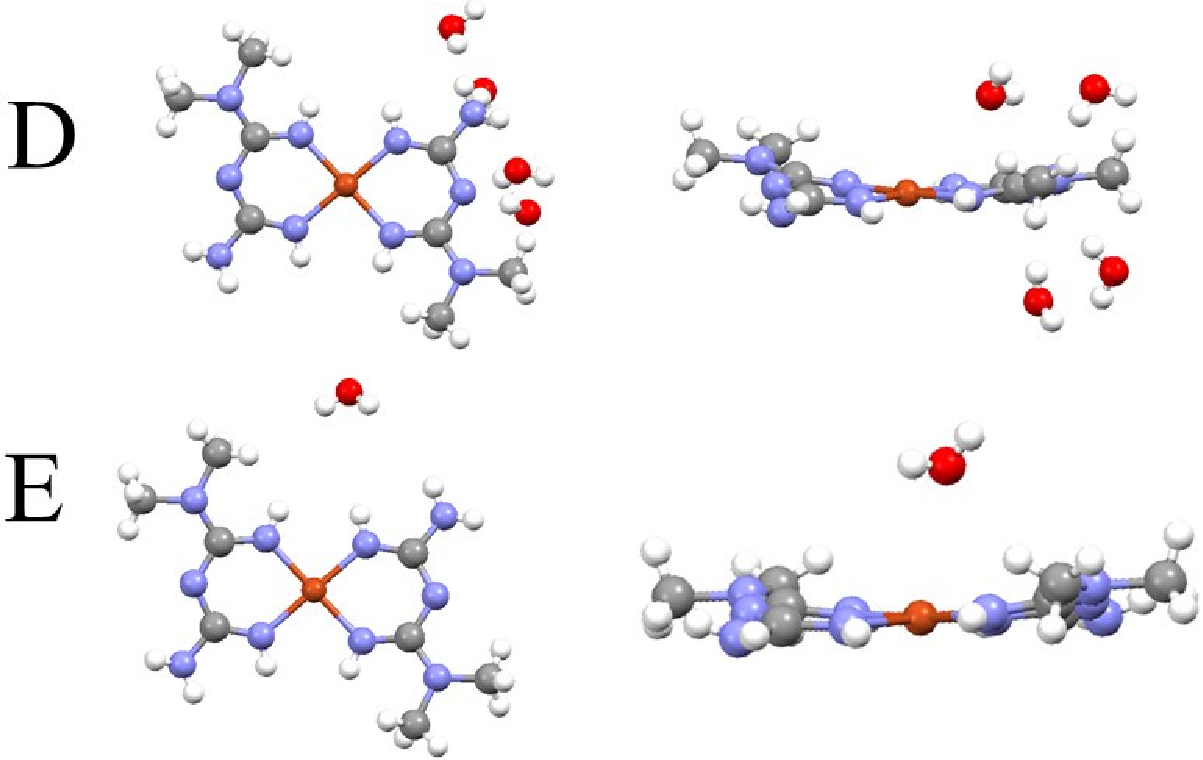


**Figure S2.** X-ray structures AJUHUJ (A), HIBPOX (B), HIHDUX (C), EFIXμM (D) and ETOFOI (E) of Cu(II)/Metformin complexes in different experimental conditions. Data available in CCDS database (https://www.ccdc.cam.ac.uk/structures/).

**SDBS-$^{1}$H NMR**SDBS No. 11236HSP-47-540
$C_{10}H_{17}N_3O_6S$
**gamma-L-glutamyl-L-cysteinylglycine**

399.65 MHz
0.041 g : 0.5 ml $D_2O$

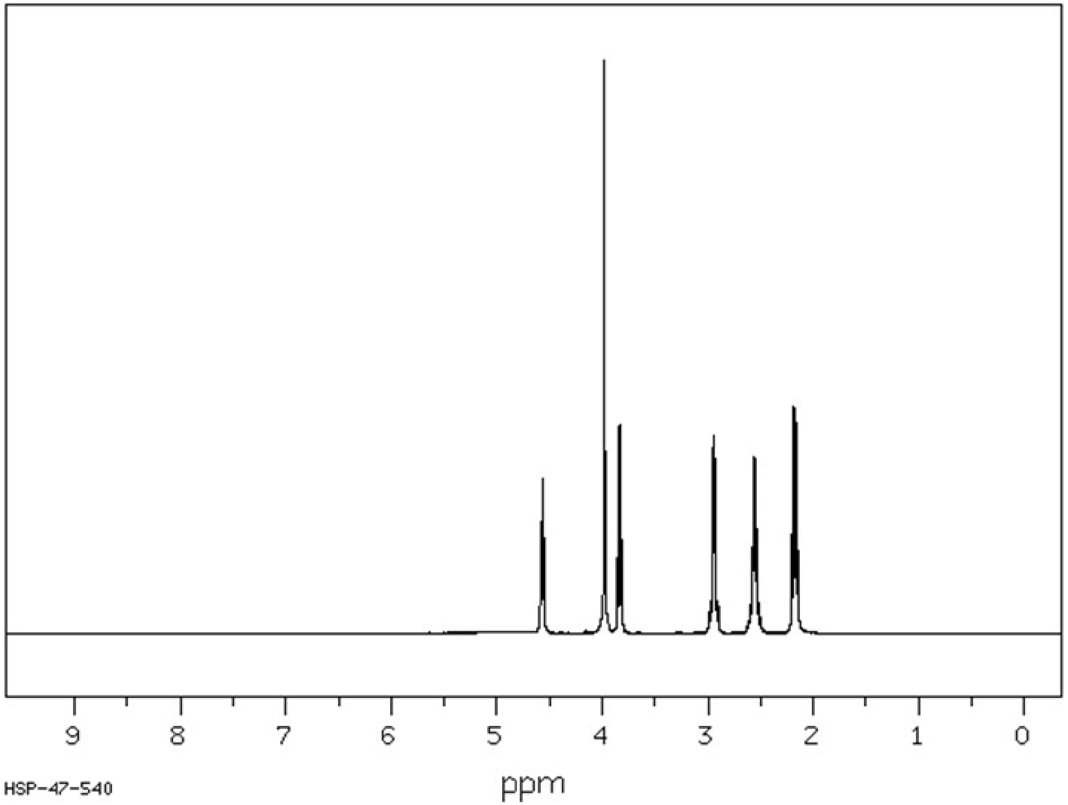


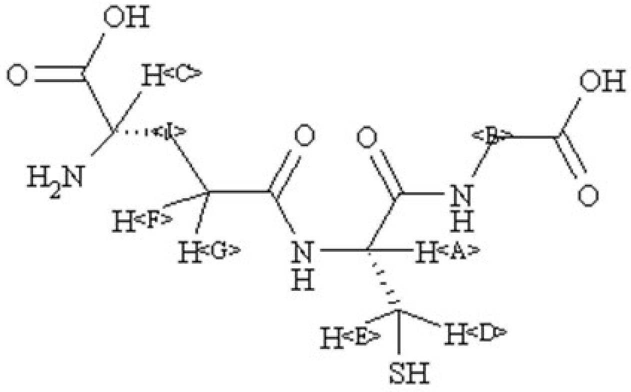


| Assign. | | Shift(ppm) |
|---|---|---|
| A | | 4.568 |
| B | | 3.978 |
| C | | 3.836 |
| D | *1 | 2.952 |
| E | *1 | 2.935 |
| F | *2 | 2.568 |
| G | *2 | 2.547 |
| J | | 2.173 |

**Figure S3.** 1HNMR spectrµM and proton assigned of GSH according to SDBS database (https://sdbs.db.aist.go.jp/sdbs/cgi-bin/cre_index.cgi).

ChemNMR $^{1}$H Estimation

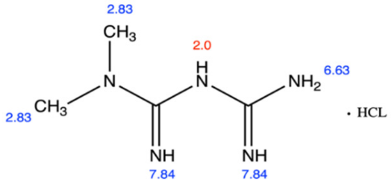


Estimation quality is indicated by color: good, medium, rough

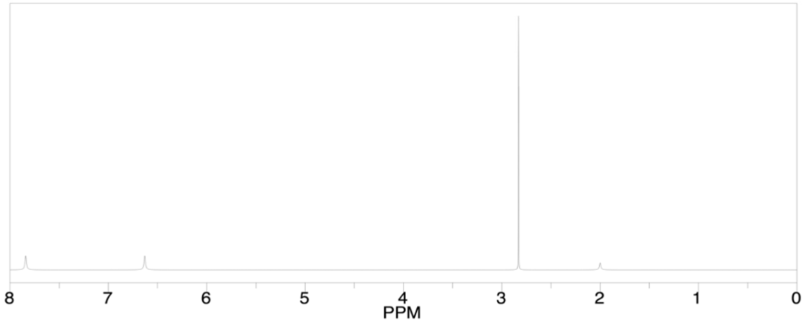


```
Protocol of the H-1 NMR Prediction (Lib=SU Solvent=DMSO 300 MHz):

Node     Shift    Base + Inc.   Comment (ppm rel. to TMS)

NH  7.84              7.50      guanidine
                         0.34      general corrections
NH  7.84              7.50      guanidine
                         0.34      general corrections
NH  2.0               2.00      guanidine
                          ?        1 unknown substituent(s) from sec. amide
                          ?        1 unknown substituent(s) from sec amine
                          ?        1 unknown substituent(s) from amine
                                   -> 2 increment(s) not found
NH2 6.63              2.00      guanidine
                         4.63      general corrections
CH3 2.83              0.86      methyl
                         1.81      1 alpha -N-C=R
                         0.16      general corrections
CH3 2.83              0.86      methyl
                         1.81      1 alpha -N-C=R
                         0.16      general corrections

1H NMR Coupling Constant Prediction

shift   atom index  coupling partner, constant and vector

7.84        5
7.84        6
2.0         2
6.63        4
2.83        8
2.83        9
```

**Figure S4.** $^{1}$H NMR prediction of metformin by Chemdraw.

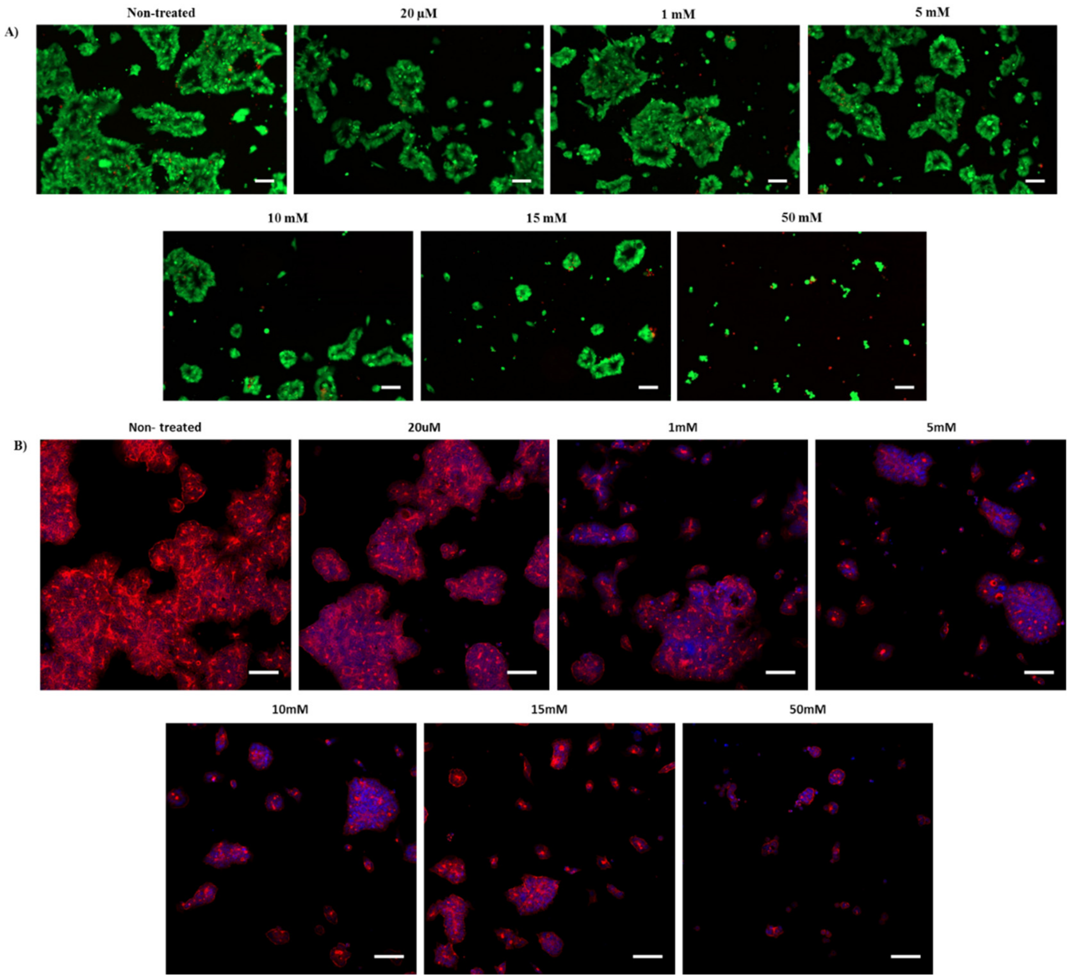


**Figure S5.** Anticancer effect of metformin on colorectal cancer (SW1222) cells. (A) Live/dead staining of SW1222 cells treated with different concentrations of metformin. The fluorescent images show live cells (green) and dead cells (red). (B) High resolution confocal images of SW1222 showing actin fibers organization (red) and nucleus (blue). Scale bar (100 µm).

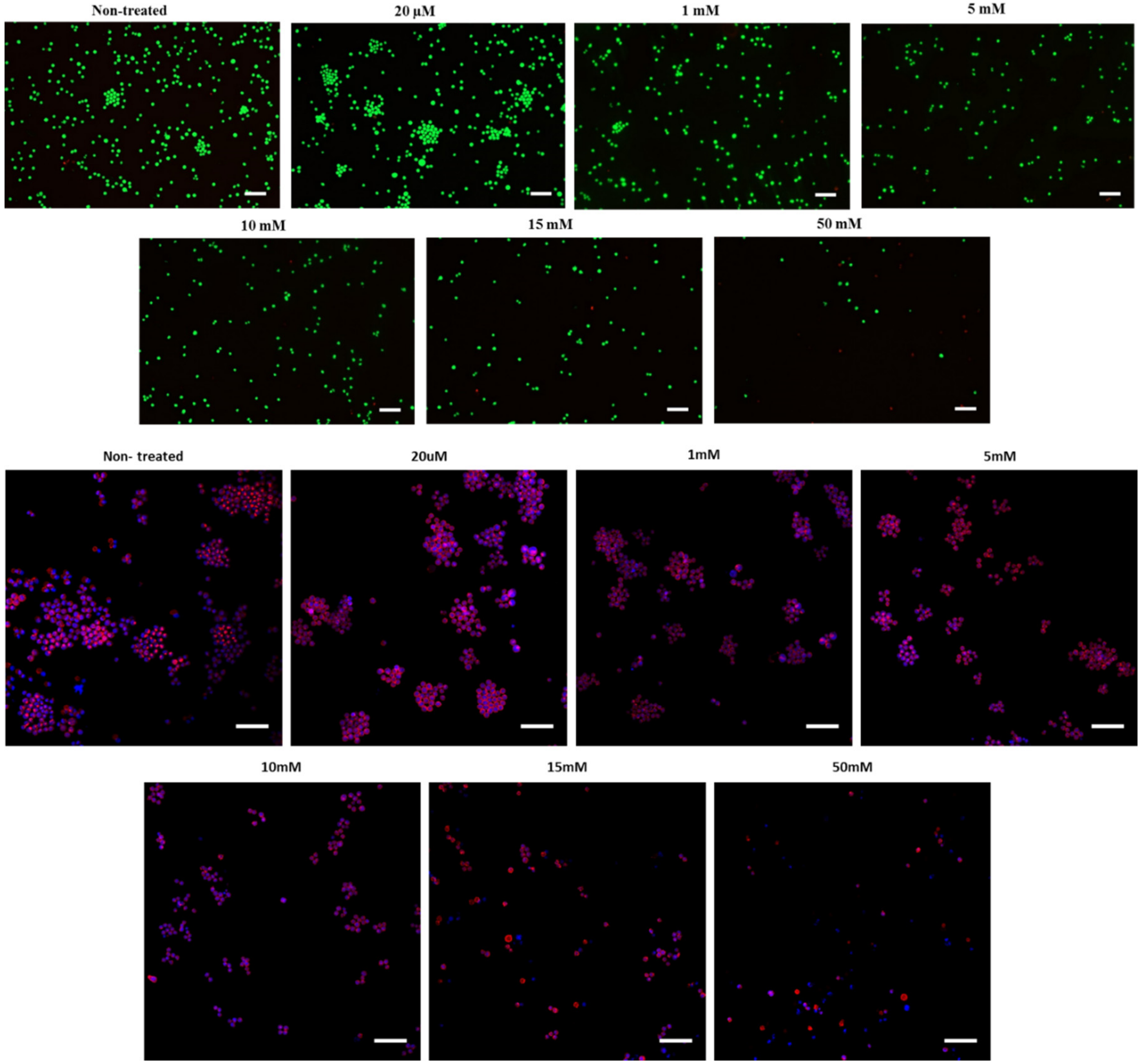


**Figure S6.** Anticancer effect of metformin on leukemic cell line (K562). (A) Live/dead staining of K562 cells treated with different concentrations of metformin. The fluorescent images show live cells (green) and dead cells (red). (B) High resolution confocal images of K652 showing actin fibers organization (red) and nucleus (blue). Scale bar (100 µm).

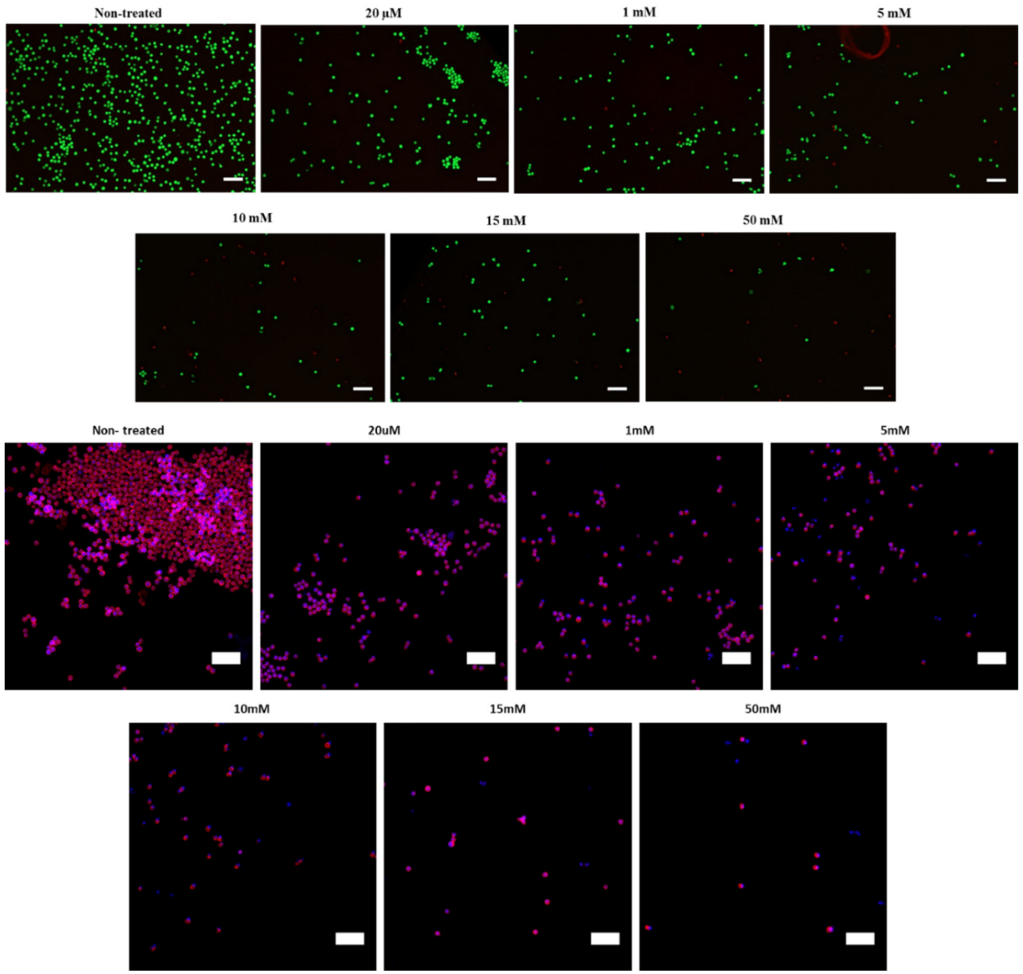


**Figure S7.** Anticancer effect of metformin on MDR leukemic cell line (K562-200). (A) Live/dead staining of K562-200 cells treated with different concentrations of metformin. The fluorescent images show live cells (green) and dead cells (red). (B) High resolution confocal images of K652-200 showing actin fibers organization (red) and nucleus (blue). Scale bar (100 µm).

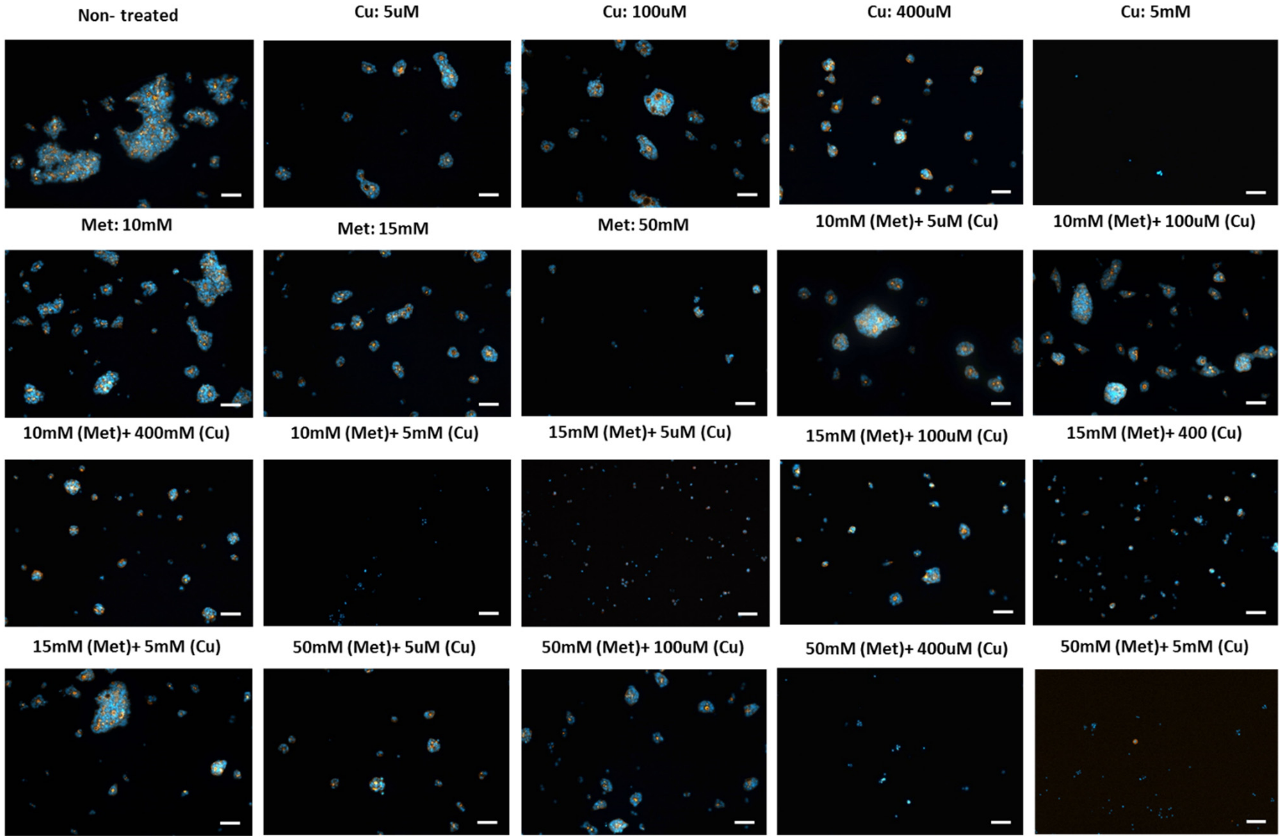


**Figure S8.** Anticancer effect of metformin on SW1222 cells in the presence and absence of copper sulfate. Scale bar (100 μM). The fluorescent images show cells shrinking and rounding with increasing concentrations of metformin with complete disappearance of actin fibers at the highest concentration used.

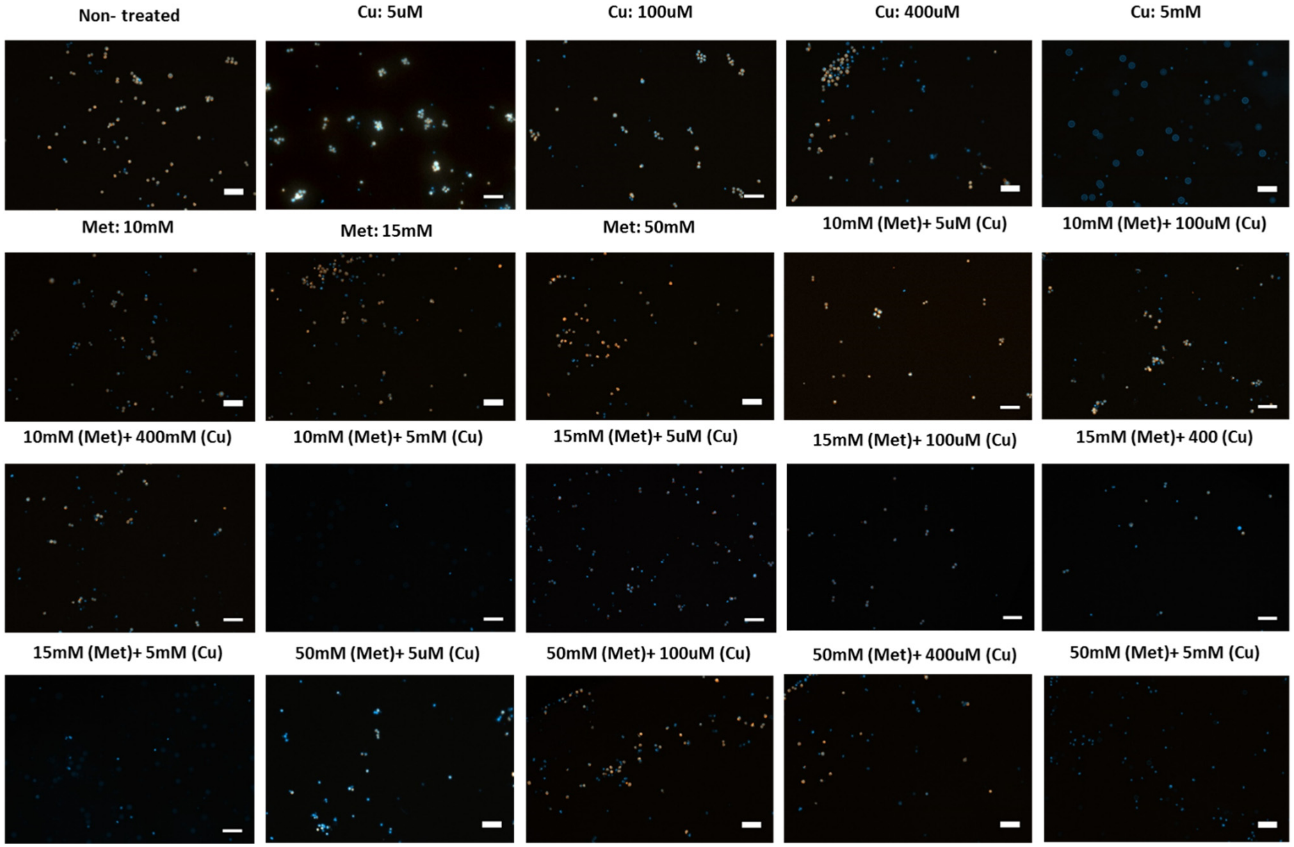


**Figure S9.** Anticancer effect of metformin on K562 cells in the presence and absence of copper sulfate. Scale bar (100 µM). The fluorescent images show cells shrinking and rounding with increasing concentrations of metformin with complete disappearance of actin fibers at the highest concentration used.

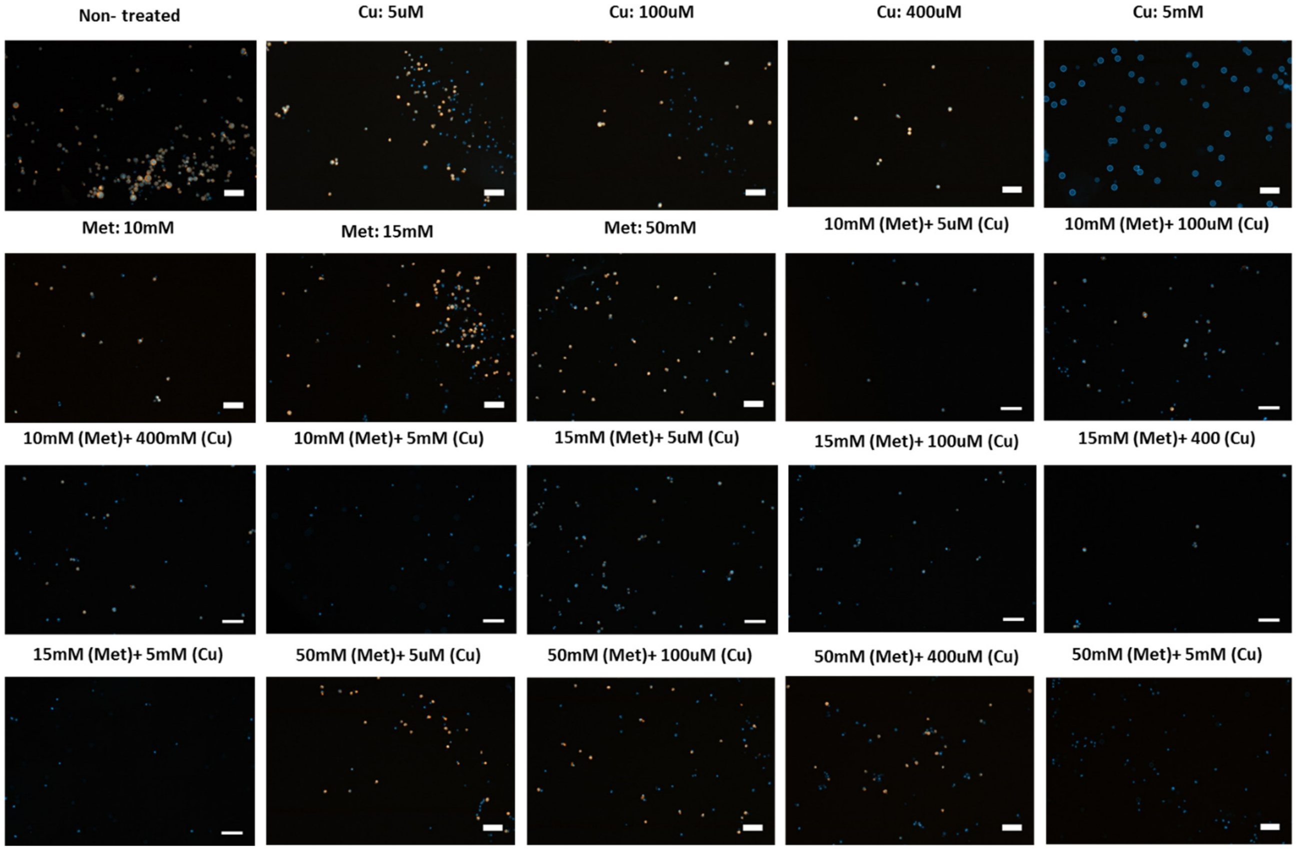


**Figure S10.** Anticancer effect of metformin on K562-200 cells in the presence and absence of copper sulfate. Scale bar (100 µM). The fluorescent images show cells shrinking and rounding with increasing concentrations of metformin with complete disappearance of actin fibers at the highest concentration used.

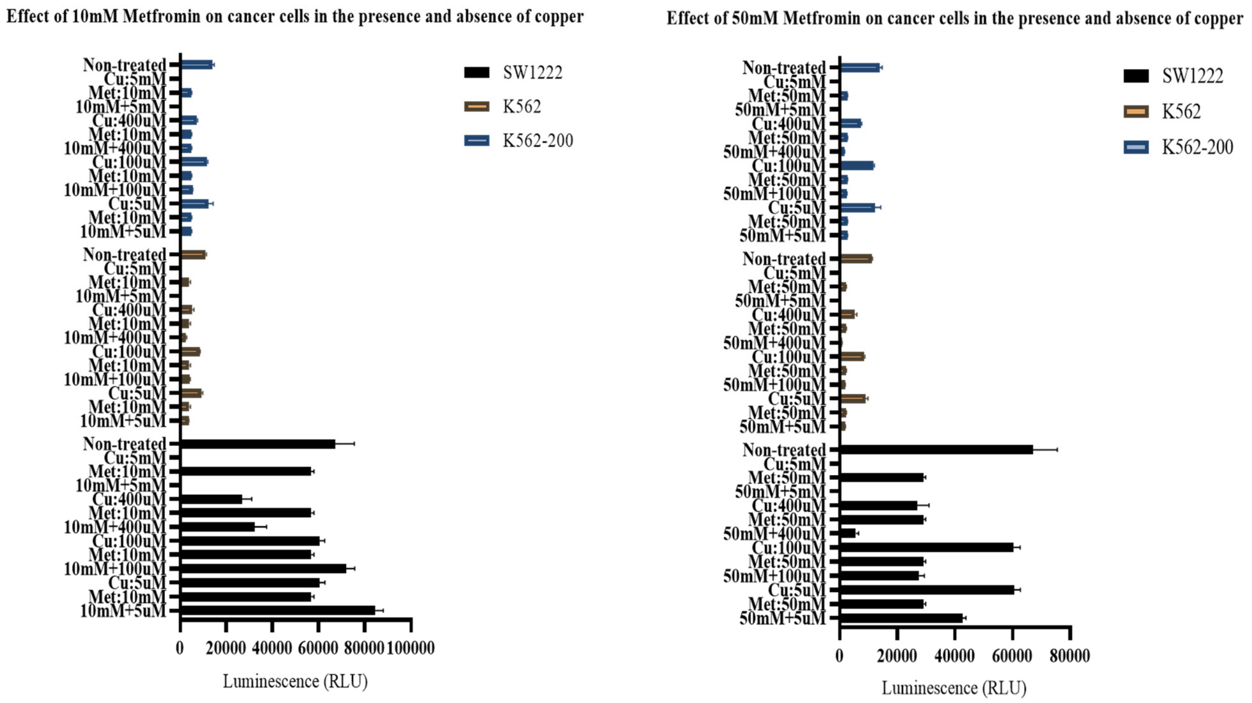


**Figure S11.** Effect of copper on the anticancer properties of metformin. Comparison between the effect on cancer cells of 10 mM (left) and 50 mM (right) metformin in the absence and presence of different concentrations of copper sulfate and the effect of copper sulfate on the same cells in the absence of metformin. (error bars different color)

**Table S1:** Effect of Cu(II) on the anticancer properties of metformin.

| Concentration (Metformin) | Concentration Cu(II) | SW1222 | | K562 | | K562-200 | |
|---|---|---|---|---|---|---|---|
| | | % of Normalized ATP Values vs. Non Treated Cells | *P* Value | % of Normalized ATP Values vs. Non Treated Cells | *P* Value | % of Normalized ATP Values vs. Non Treated Cells | *P* Value |
| 10 mM | 5 μM | 126 | 0.05 | 33.5 | <0.001 | 34.8 | 0.002 |
| 10 mM | 100 μM | 107.2 | 0.4 | 37.7 | <0.001 | 38.7 | 0.003 |
| 10 mM | 400 μM | 48 | 0.006 | 22.5 | <0.001 | 34 | 0.001 |
| 10 mM | 5 mM | 0.27 | 0.005 | 0.36 | <0.001 | 0.34 | 0.001 |
| 15 mM | 5 μM | 112.5 | 0.2 | 41.2 | <0.001 | 34.1 | 0.003 |
| 15 mM | 100 μM | 83.4 | 0.2 | 33.5 | <0.001 | 35 | 0.003 |
| 15 mM | 400 μM | 37.9 | 0.01 | 19.6 | <0.001 | 15.3 | <0.001 |
| 15 mM | 5 mM | 0.12 | 0.005 | 0.32 | <0.001 | 0.27 | 0.001 |
| 50 mM | 5 μM | 63.5 | 0.03 | 17.5 | <0.001 | 19.7 | 0.002 |
| 50 mM | 100 μM | 40.9 | 0.01 | 16.9 | <0.001 | 17.9 | 0.002 |
| 50 mM | 400 μM | 8.3 | 0.006 | 6.8 | <0.001 | 11.8 | 0.002 |
| 50 mM | 5 mM | 0.16 | 0.005 | 0.32 | <0.001 | 0.27 | 0.001 |
| 10 mM | 0 | 84.6 | 0.2 | 35.2 | <0.001 | 34 | 0.001 |
| 15 mM | 0 | 71.2 | 0.04 | 34.9 | <0.001 | 27.3 | 0.003 |
| 50 mM | 0 | 43.4 | 0.02 | 20.2 | <0.001 | 19.5 | 0.002 |
| 0 | 5 μM | 90.1 | 0.3 | 81.8 | 0.03 | 88.5 | 0.3 |
| 0 | 100 μM | 89.9 | 0.3 | 76.5 | <0.001 | 84.9 | 0.05 |
| 0 | 400 μM | 39.9 | 0.005 | 47.2 | 0.002 | 52.7 | 0.003 |
| 0 | 5 mM | 0.12 | 0.005 | 0.31 | <0.001 | 0.31 | 0.001 |

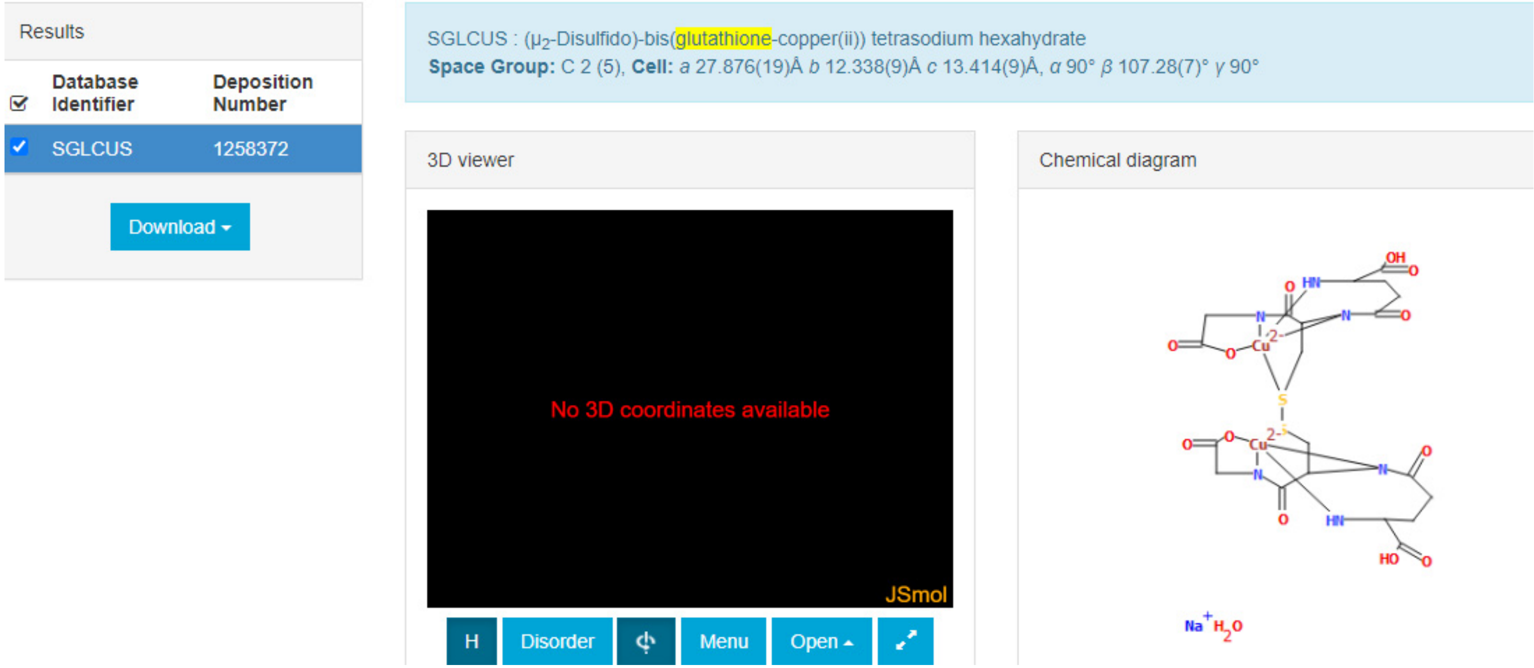


**Figure S12.** Chemical diagram of the X-ray structures of Cu(II)/GSSG complex [1]. Data available in CCDS database (https://www.ccdc.cam.ac.uk/structures/).

1 **Miyoshi, K., Sugiura, Y., Ishizu, K., Iitaka, Y. & Nakamura, H. Crystal structure and spectroscopic properties of violet glutathione-copper (II) complex with axial sulfur coordination and two copper sites via a disulfide bridge. *Journal of the American Chemical Society* 102, 6130-6136 (1980).**
